# End-stage renal disease, calcification patterns and clinical outcomes after TAVI

**DOI:** 10.1007/s00392-021-01968-y

**Published:** 2021-11-13

**Authors:** David Grundmann, Matthias Linder, Alina Goßling, Lisa Voigtländer, Sebastian Ludwig, Lara Waldschmidt, Till Demal, Oliver D. Bhadra, Andreas Schäfer, Johannes Schirmer, Hermann Reichenspurner, Stefan Blankenberg, Dirk Westermann, Niklas Schofer, Lenard Conradi, Moritz Seiffert

**Affiliations:** 1 Department of Cardiology, University Heart and Vascular Center Hamburg, Martinistraße 52, 20251 Hamburg, Germany; 2 German Center for Cardiovascular Research (DZHK), Partner Site Hamburg/Lübeck/Kiel, Hamburg, Germany; 3 Department of Cardiovascular Surgery, University Heart and Vascular Center Hamburg, Martinistraße 52, 20251 Hamburg, Germany

**Keywords:** CKD, ESRD, TAVI, Vascular calcification, AVC

## Abstract

**Background:**

Patients with chronic hemodialysis due to end-stage renal disease (ESRD) or severely impaired kidney function (CKD) constitute a relevant share of patients undergoing trans-catheter aortic valve implantation (TAVI). However, data on specific challenges and outcomes remain limited.

**Aim:**

We aimed to characterize this patient population, evaluate clinical results and assess the significance of calcification patterns.

**Methods:**

This retrospective single-center analysis evaluated 2,712 TAVI procedures (2012–2019) according to baseline renal function: GFR < 30 ml/min/1.73m^2^ (CKD; *n* = 210), chronic hemodialysis (ESRD; *n* = 119) and control (CTRL; *n* = 2383). Valvular and vascular calcification patterns were assessed from contrast-enhanced multi-detector computed tomography. Outcomes were evaluated in accordance with the VARC-2 definitions.

**Results:**

Operative risk was higher in ESRD and CKD vs. CTRL (STS-score 8.4% and 7.6% vs. 3.9%, *p* < 0.001) and patients with ESRD had more severe vascular calcifications (49.1% vs. 33.9% and 29.0%, *p* < 0.01). Immediate procedural results were similar but non-procedure-related major/life-threatening bleeding was higher in ESRD and CKD (5.0% and 5.3% vs. 1.6%, *p* < 0.01). 3-year survival was impaired in patients with ESRD and CKD (33.3% and 35.3% vs. 65.4%, *p* < 0.001). Multivariable analysis identified ESRD (HR 1.60), CKD (HR 1.79) and vascular calcifications (HR 1.29) as predictors for 3-year and vascular calcifications (HR 1.51) for 30-day mortality.

**Conclusion:**

Patients with ESRD and CKD constitute a vulnerable patient group with extensive vascular calcifications. Immediate procedural results were largely unaffected by renal impairment, yielding TAVI a particularly valuable treatment option in these high-risk operative patients. Mid-term survival was determined by underlying renal disease, cardiovascular comorbidities, and vascular calcifications as a novel risk marker.

**Graphical abstract:**

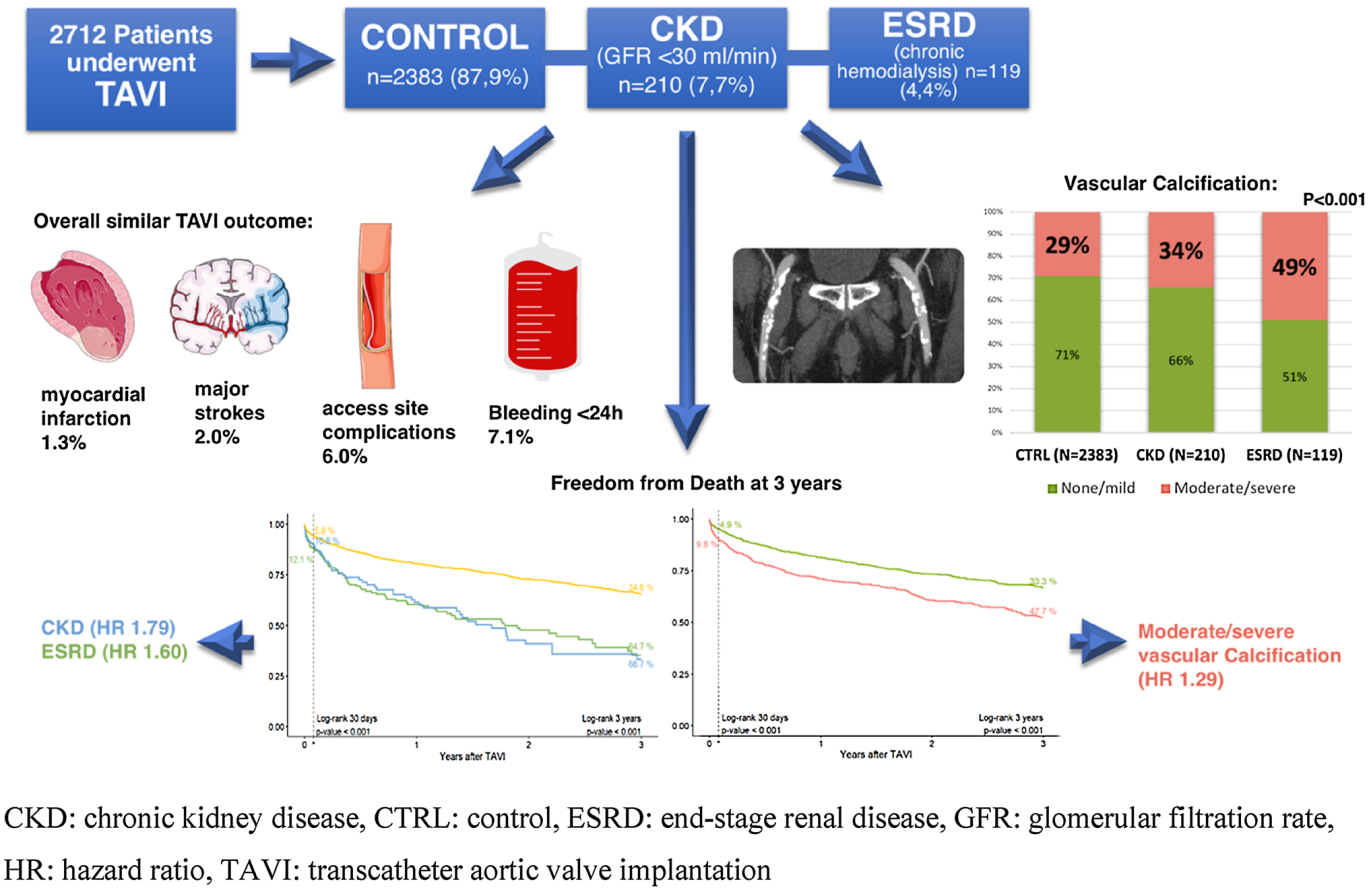

**Supplementary Information:**

The online version contains supplementary material available at 10.1007/s00392-021-01968-y.

## Introduction

Aortic stenosis (AS) remains the most common valvular heart disease in Europe with significant impact on morbidity and mortality. Surgical aortic valve replacement (SAVR) was considered the standard of care for decades. However, transcatheter aortic valve implantation (TAVI) has meanwhile become the preferred treatment option for patients with severe AS at intermediate or high operative risk [1–3]. Moreover, non-inferiority to SAVR has recently been demonstrated in low-risk patients [2–4].

Comorbidities in patients with AS are frequent and prevalence of chronic kidney disease (CKD) or end-stage renal disease (ESRD) are relevant in this population: Severe CKD [glomerular filtration rate (GFR) < 30 ml/min] is reported in 5 to 8% and ESRD in 4% of patients in registries and clinical trials [5–11]. Main risk factors include hypertension and diabetes leading to renal fibrosis [12]. Furthermore, CKD and ESRD are associated with an earlier and faster progression of AS severity and severe vascular calcifications [13–15]. Impaired outcomes were reported after TAVI [10, 16]. However, due to exclusion from most randomized trials, procedural characteristics, mid-term outcomes and data on specific challenges in patients with severe CKD and ESRD remain limited.

Aims of our study were (i) to determine the prevalence of ESRD (chronic hemodialysis) or severe CKD among patients scheduled for TAVI and characterize this patient population, (ii) to assess vascular and valvular calcification patterns in these patients, (iii) to evaluate immediate procedural results and challenges in this patient group, and (iv) to investigate the impact of renal function on mid-term outcomes.

## Methods

### Patient population

From January 2012 until August 2019, 2,712 consecutive patients underwent TAVI at the University Heart and Vascular Center Hamburg and were included in this single-center retrospective analysis to reflect clinical reality. All patients provided informed consent to the procedure and data acquisition. Ethics committee approval was obtained according to local requirements. The study was performed in accordance with the 1964 Declaration of Helsinki and its later amendments.

GFR was calculated according to the Chronic Kidney Disease Epidemiology Collaboration (CKD-EPI) equation [17]. Eight patients were excluded from analysis due to missing data on baseline renal function. The study population was stratified into three groups according to baseline kidney function: GFR < 30 ml/min (CKD; *n* = 210), end-stage renal disease requiring chronic hemodialysis (ESRD; *n* = 119) and control group (CTRL; *n* = 2383).

### Clinical outcomes and endpoint definitions

All cases were reviewed by the local heart team and agreed to be eligible for TAVI. Periprocedural results and clinical outcomes were consecutively assessed according to the updated Valve Academic Research Consortium definitions [18].

### Computed tomography assessment

**Fig. 1:**
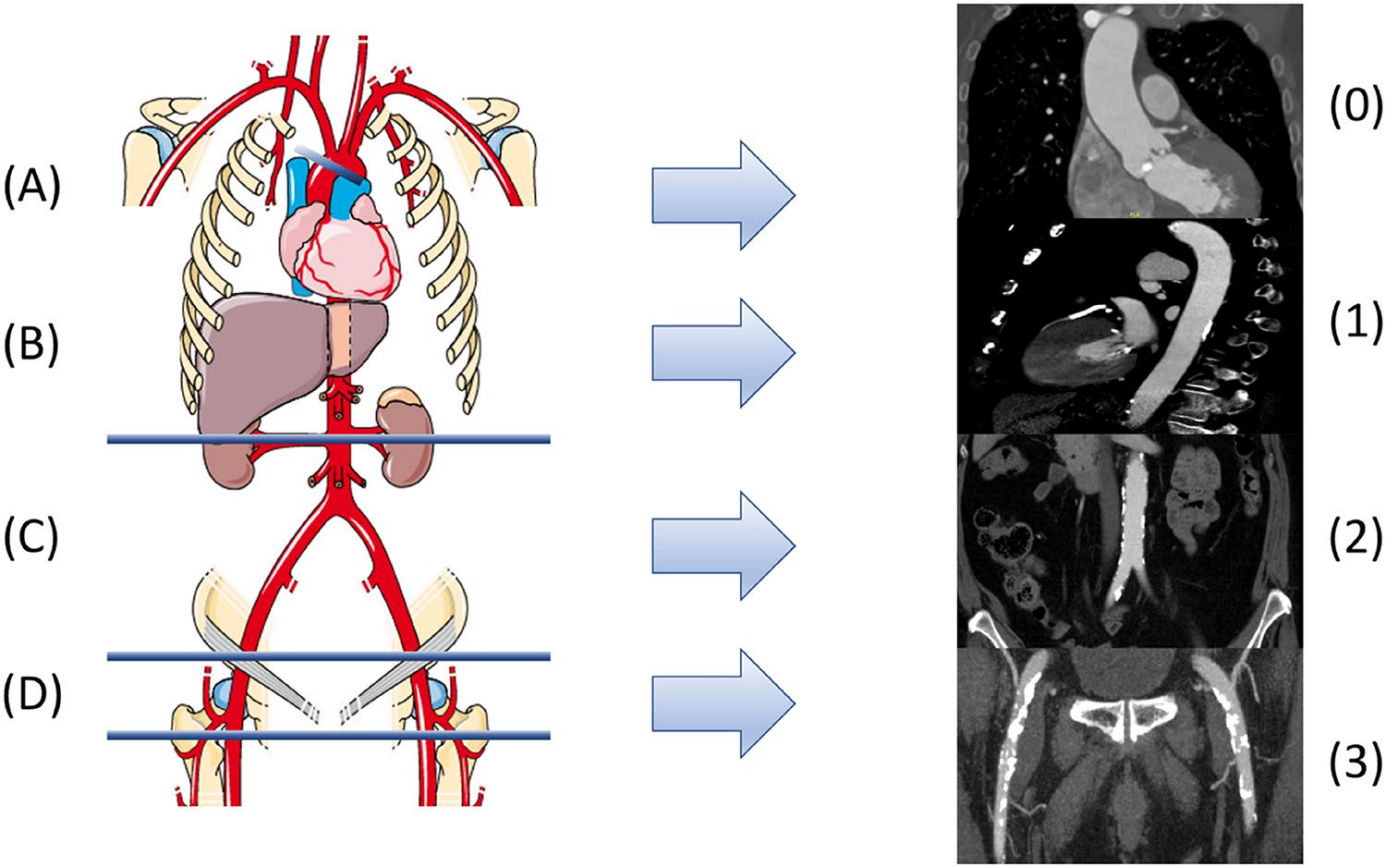
Arterial segmentation and vascular calcification severity in contrast-enhanced MDCT images. **A** Ascending aorta, **B** descending aorta, **C** infrarenal aorta/iliac, **D** femoral arteries. VCSS: 0: no calcification 1: mild calcification 2: moderate calcification, 3: severe calcification. MDCT: multi-detector computed tomography, VCSS: vascular calcification severity score. Image Source: https://smart.servier.com/wp-content/uploads/2016/10/Systeme_arteriel_2.png

Routine contrast-enhanced multi-detector computed tomography (MDCT) was performed during pre-TAVI workup. Dimensions of the aortic annulus and root and calcification of the aortic valve complex were assessed with the 3-Mensio Structural Heart Software V9.1 (Pie Medical Imaging, Maastricht, Netherlands) [19]. Valvular calcium burden was assessed in contrast-enhanced MDCT images using a volume-scoring tool. Calcium detection threshold was set at 500 Hounsfield units (HU) for discrimination against contrast medium, as previously described [19]. Vascular calcification severities and patterns were evaluated for 2,639 patients in a semi-quantitative and blinded fashion. The arterial vascular system was divided into 4 segments: 1. ascending aorta, (annular plane to brachiocephalic trunk), 2. descending aorta (aortic arch to takeoff of renal arteries), 3. infra-renal aorta/iliac arteries (takeoff of renal arteries to inguinal ligament), and 4. femoral arteries (inguinal ligament to femoral artery bifurcation) (Fig. 1). Calcification severity was graded from 0 to 3 according to a Vascular Calcification Severity Score (VCSS) for each segment: (0) Complete absence of calcification at the respective segment, (1) calcification of < 10% of the target segment length, (2) calcification of ≥ 10% and ≤ 50% of the target segment length and (3) calcium distribution to > 50% of the target segment length. VCSS for all 4 segments were added to assess overall vascular calcification and defined as moderate/severe (VCSS ≥ 7, *n* = 797) and none/mild (VCSS ≤ 6, *n* = 1842).

### Statistical analysis

Binary variables were shown as absolute numbers or percentages and were compared using the c^2^ test. Continuous variables were shown as median (interquartile range) and were compared using the Kruskal–Wallis or Mann–Whitney tests, as appropriate. Survival curves for all-cause mortality were produced using the Kaplan–Meier method. The log-rank test was used to test for survival curve differences. Mortality predictors were assessed using univariable and multivariable Cox regression analyses through a best performing selection process. Renal function (ESRD vs. CKD vs. CTRL) was forced into the model along with age, male sex, body mass index (BMI), diabetes, New York Heart Association class I–III and IV, coronary artery disease, prior myocardial infarction, prior coronary artery bypass grafting, cerebrovascular accident, extra-cardiac Arteriopathy, pulmonary hypertension, prior atrial fibrillation, anemia, chronic obstructive pulmonary disease (COPD), ejection fraction < 30%, implant approach (non-TF versus TF), and high vascular calcification overall. All p values had a significance threshold of < 0.05. Statistical analyses were performed using R version 4.0.3 (R Foundation for Statistical Computing).

## Results

### Baseline characteristics

ESRD and CKD were present in 4.4% and 7.7% of patients, respectively. Patients with ESRD were younger, with a lower body mass index and predominantly male compared to CKD and CTRL groups (Table 1). Prevalences of impaired left-ventricular function and peripheral artery disease were increased in this group. Rates of prior myocardial infarction, NYHA IV, severe pulmonary hypertension, and concomitant mitral or tricuspid regurgitation were higher in both ESRD and CKD patients. Common comorbidities in these two groups included anemia and diabetes. The predicted operative risk was elevated, accordingly (Table 1). For baseline characteristics according to vascular calcification severity, see Online Resource 1.

**Table 1:** Baseline and procedural characteristics

	All (*N* = 2712)	CKD (*N* = 210)	ESRD (*N* = 119)	CTRL (*N* = 2383)	*p* value
Sex (male) (%)	1284 (47.3)	69 (32.9)	84 (70.6)	1131 (47.5)	** < 0.001**
Age (years)	81.0 (76.5, 84.8)	83.4 (79.2, 87.5)	75.1 (68.9, 80.2)	81.0 (76.7, 84.8)	** < 0.001**
BMI (kg/m^2^)	26.2 (23.4, 29.7)	27.0 (24.3, 29.9)	24.7 (22.7, 29.1)	26.1 (23.4, 29.7)	**0.022**
NYHA class IV (%)	352 (13.2)	42 (20.2)	25 (21.4)	285 (12.2)	** < 0.001**
LVEF < 30% (%)	291 (11.0)	26 (12.7)	23 (19.8)	242 (10.4)	**0.004**
Mean transvalvular gradient (mmHg)	33.0 (23.0, 44.0)	31.0 (22.0, 42.0	27.5 (19.9, 40.1)	33.0 (23.0, 45.0)	**0.001**
Effective orifice area (cm^2^)	0.8 (0.6, 0.9	0.8 (0.6, 0.9)	0.8 (0.7, 1.0	0.8 (0.6, 0.9	0.14
Mitral regurgitation ≥ moderate (%)	881 (33.4)	96 (46.4)	55 (47.0)	730 (31.5)	** < 0.001**
Tricuspid regurgitation ≥ moderate (%)	615 (24.1)	63 (32.1)	43 (38.1)	509 (22.7)	** < 0.001**
Pulmonary hypertension (PAP syst > 55 mmHg (%)	349 (19.2)	35 (25.4)	25 (30.5)	289 (18.1)	**0.003**
Diabetes mellitus (%)	773 (28.5)	76 (36.2)	42 (35.3)	655 (27.5)	**0.007**
Atrial fibrillation (%)	792 (30.2)	72 (35.5)	39 (33.9)	681 (29.5)	0.14
PAD (%)	789 (29.1)	68 (32.4)	47 (39.5)	674 (28.3)	**0.018**
Prior PM / ICD (%)	293 (10.8)	36 (17.2)	13 (11.0)	244 (10.3)	**0.008**
Coronary artery disease (%)	1735 (64.4)	137 (65.6)	84 (71.2)	1514 (64.0)	0.26
Prior myocardial infarction (%)	390 (14.4)	42 (20.0)	24 (20.2)	324 (13.6)	**0.007**
Prior PCI (%)	993 (36.8)	76 (36.7)	52 (44.1)	865 (36.4)	0.24
Prior CABG (%)	381 (14.1)	34 (16.3)	21 (17.8)	326 (13.7)	0.29
Prior stroke (%)	405 (14.9)	33 (15.7)	25 (21.0)	347 (14.6)	0.15
COPD (%)	505 (18.6)	27 (12.9)	22 (18.5)	456 (19.1)	0.081
Anemia (hemoglobin < 11 g/dl) (%)	793 (29.3)	112 (53.3)	75 (63.0)	606 (25.5)	** < 0.001**
GFR (CKD-EPI) (mL/min/1.73m^2^)	57.0 (40.9, 74.1)	24.5 (19.8, 28.0)	10.2 (7.4, 16.1)	60.7 (47.0, 76.5)	** < 0.001**
STS PROM (%)	4.2 (2.7, 6.5)	7.6 (5.4, 11.6)	8.4 (5.7, 13.1)	3.9 (2.6, 5.8)	** < 0.001**
Logistic Euro-Score II (%)	4.4 (2.6, 7.7)	7.2 (4.8, 13.2)	6.0 (3.4, 11.6)	4.1 (2.4, 7.1)	** < 0.001**
Transfemoral access (%)	2089 (77.0)	165 (78.6)	84 (70.6)	1840 (77.2)	0.21
THV					
Balloon-expandable (%)	1093 (40.3)	88 (41.9)	42 (35.3)	963 (40.4)	0.48
Mechanically- and self- expanding (%)	1619 (59.7)	122 (58.1)	77 (64.7)	1420 (59.6)	0.48
Conscious sedation (%)	1410 (52.3)	101 (48.1)	52 (44.4)	1257 (53.0)	0.088
Procedure time (min)	80.0 (65.0, 105.0)	85.0 (65.0, 105.3)	85.0 (70.0, 110.4)	80.0 (65.0, 105.0)	0.12
Contrast (mL)	163.0 (120.0, 212.0)	140.0 (97.8, 193.0)	154.0 (120.4, 202.9)	165.5 (122.9, 215.0)	** < 0.001**
Fluoroscopy time (min)	16.2 (10.7, 24.0)	16.1 (10.9, 26.4)	15.8 (10.5, 23.8)	16.3 (10.7, 23.9)	0.58
THV size (mm)	26.0 (23.0, 27.0)	26.0 (23.0, 27.0)	27.0 (25.9, 29.0)	26.0 (23.0, 27.0)	** < 0.001**
Pre-dilation (%)	1933 (71.5)	136 (65.1)	83 (69.7)	1714 (72.2)	0.083
Post-dilation (%)	960 (35.7)	76 (36.7)	44 (37.3)	840 (35.5)	0.88
Length of ICU stay (days)	1.0 (1.0, 2.0)	2.0 (1.0, 4.0)	2.0 (1.0, 3.0)	1.0 (1.0, 2.0)	** < 0.001**
Length of hospital stay (days)	8.0 (7.0, 11.0)	9.0 (7.0, 15.0)	9.0 (7.0, 14.6)	8.0 (7.0, 11.0)	** < 0.001**

Values are *n* (%) or median (interquartile range)

*BMI* body mass index, *CABG* coronary artery bypass grafting, *CAD* coronary artery disease, *CKD* chronic kidney disease, *COPD* chronic obstructive pulmonary disease, *CTRL* Control, *ESRD* end-stage renal disease, *GFR* glomerular filtration rate, *ICD* implantable cardioverter defibrillator, *ICU* intensive care unit, *LVEF* left-ventricular ejection fraction, *NYHA* New York Heart Association, *PAD* peripheral artery disease, *PAP* pulmonary artery pressure, *PCI* percutaneous coronary interventions, *PM* pacemaker, *STS-PROM* Society of Thoracic Surgeons Predicted Risk of Mortality, *THV* transcatheter heart valve

Bold values indicate statistical significance as defined by *p* < 0.05

**Fig. 2:**
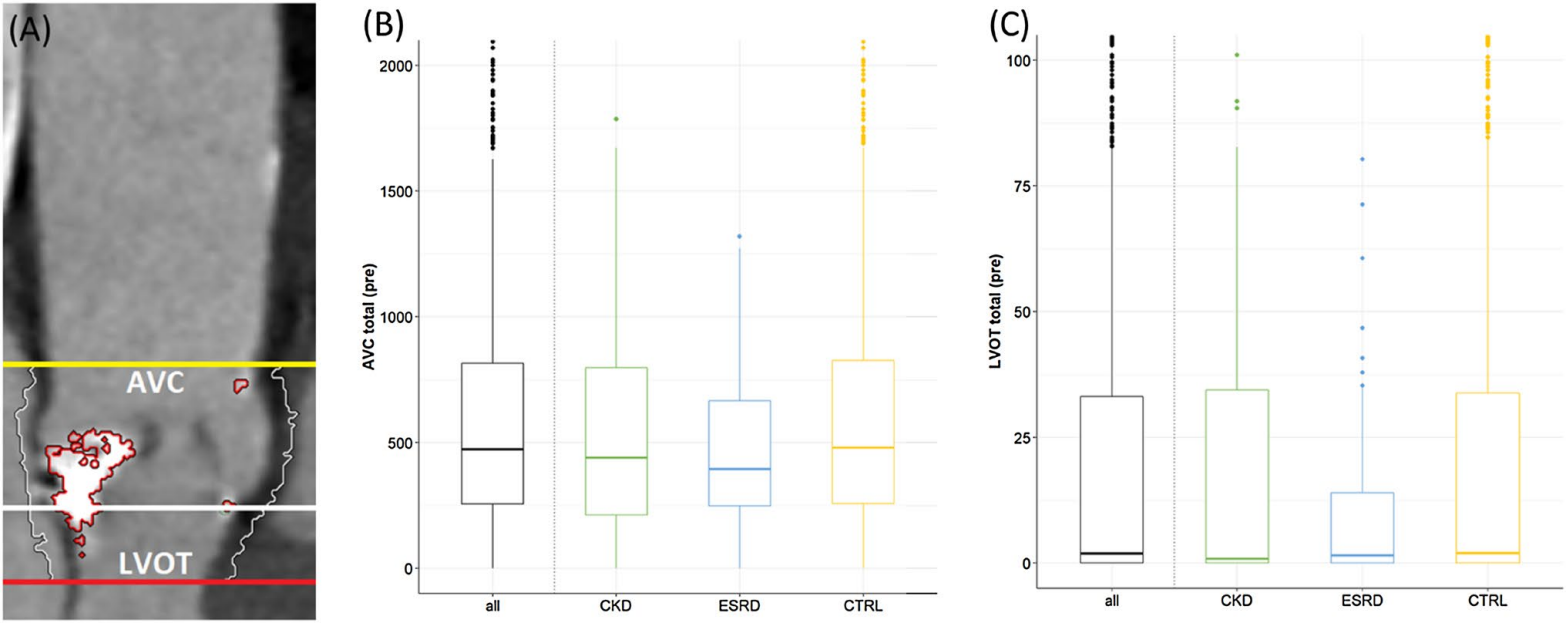
Baseline measurements of AVC and LVOT calcification in contrast-enhanced MDCT images. **A** LVOT and AVC calcification quantification. The aortic annulus is represented by the virtual basal plane indicating the basal attachment of the three cusps (white line). The AVC was defined from basal plane to coronary ostia (white to yellow line); the LVOT was defined 10 mm inferior to the basal plane (white to red line). **B** Baseline MDCT-based measurements of AVC Calcification in mm^3^ according to renal function. **C** Baseline MDCT-based measurements of LVOT Calcification in mm^3^ according to renal function. *AVC* aortic valve calcification, *CKD* chronic kidney disease, *CTRL* control, *ESRD* end-stage renal disease, *LC* left coronary, *LVOT* left ventricular outflow tract, *MDCT* multi-detector computed tomography, *NC* non-coronary, *RC* right coronary

**Fig. 3:**
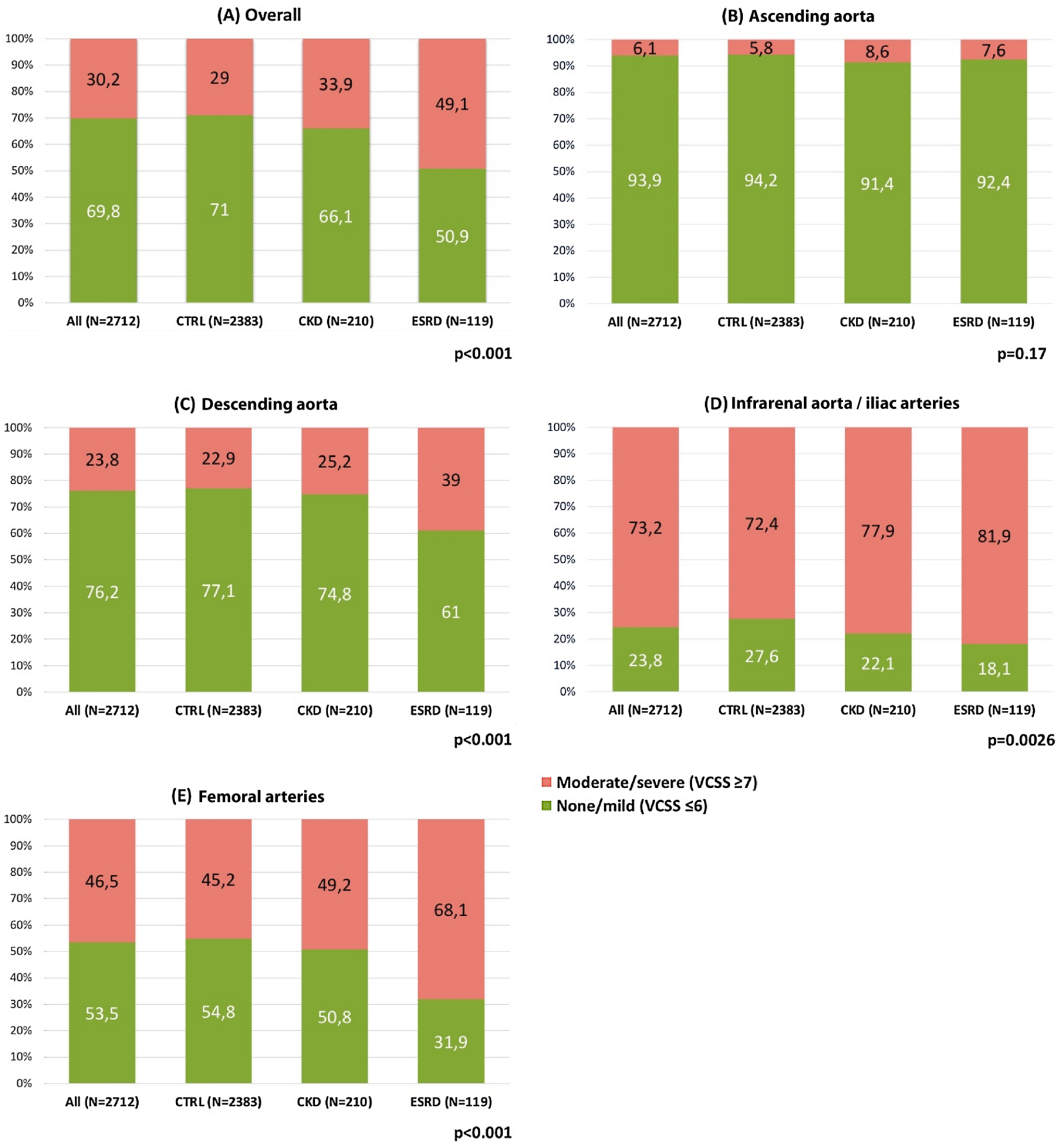
Prevalence and severity of vascular calcification according to renal function. **A** All segments, **B** Ascending aorta, **C** Descending aorta, **D** Infrarenal aorta/iliac arteries, **E** Femoral arteries. *CKD* chronic kidney disease, *CTRL* control, *ESRD* end-stage renal disease, *VCSS* vascular calcification severity score

Annulus and LVOT perimeters were larger in ESRD compared to CKD and CTRL patients (Online Resource 2). Calcification severity of the aortic valve complex and left ventricular outflow tract was similar but vascular calcification was observed more often in ESRD compared to CKD and CTRL patients (moderate/severe; 49.1% vs. 33.9% and 29.0%, *p* < 0.01, Figs. 2 and 3). While calcium burden was similar in the ascending aorta, it was significantly higher in the descending aorta (39.0% vs. 25.3% and 22.9%), infrarenal aorta/iliac (81.9% vs. 77.9% vs. 72.4%) and femoral arteries (68.1% vs. 49.3% and 45.2%) in ESRD vs. CKD and CTRL patients (all *p* < 0.05). Distribution of moderate/severe calcification across all four vascular segments was similar among all groups with the major burden in the infrarenal aorta/iliac, followed by femoral arteries, descending and ascending aorta.

### Procedural parameters and clinical outcomes

Rates of non-trans-femoral access did not differ significantly and use of contrast agent was lowest in CKD patients. Apart from that, TAVI was performed with similar procedural characteristics, technical aspects, and functional results among patients with ESRD, CKD and CTRL (Table 1). Balloon-expandable and self-expanding or mechanically expanding THV were implanted in 40.3 and 59.7%, respectively. Most frequent THV types included Sapien XT / Sapien 3 (40.3%, Edwards Lifesciences, Irvine, USA), Acurate / Acurate Neo (23.1%, Symetis/Boston Scientific, Marlborough, USA), and CoreValve Evolut / Evolut R (8.7%, Medtronic Inc., Minnesota, USA). THV types did not differ substantially between the groups (Online Resource 3).

Patients with ESRD and CKD spent longer time in the intensive care units and in-hospital. Myocardial infarction (1.3%), major stroke (2.0%), and minor (9.6%) or major access-site complications (6.0%) were similar among all groups at 30 days (Table 2). Major/life-threatening bleeding was most prevalent in patients with CKD (22.7% vs. 16.0% vs. 12.4%, *p* < 0.001). With similar event rates in the early post-procedural phase < 24 h after TAVI, an increased frequency of major/life-threatening bleeding was observed in CKD and ESRD patients thereafter (> 24 h after TAVI; 13.5% and 10.1% vs. 5.4%, *p* < 0.001). Acute renal failure occurred more often in CKD patients compared to CTRL (AKIN ≥ 2; 11.6% vs. 4.2%, p < 0.001). Permanent pacemaker implantation was performed significantly more often in patients with ESRD compared to CKD and CTRL (25.2% vs. 12.1% and 14.9%, *p* = 0.004), mostly due to third-degree atrioventricular block (AVB; 70.5%) which were observed significantly more frequent in case of ESRD (17.6%) compared to CKD (8.6%) and CTRL (10.3%, *p* = 0.040, respectively).

**Table 2:** Procedural complications and 30-day outcomes after TAVI according to renal function and vascular calcification

	Renal function					Vascular calcification			
	All (*N* = 2712)	CKD (*N* = 210)	ESRD (*N* = 119)	CTRL (*N* = 2383)	*p* value	All (*N* = 2639)	None/mild (*N* = 1842)	Moderate/ severe (*N* = 797)	*p* value
Myocardial infarction (%)	34 (1.3)	5 (2.4)	1 (0.8)	28 (1.2)	0.30	33 (1.3)	23 (1.3)	10 (1.3)	1.00
Any stroke/TIA (%)	105 (3.9)	10 (4.8)	3 (2.5)	92 (3.9)	0.60	103 (3.9)	69 (3.7)	34 (4.3)	0.60
Disabling stroke (%)	54 (2.0)	5 (2.4)	2 (1.7)	47 (2.0)	0.89	54 (2.0)	32 (1.7)	20 (2.5)	0.25
Major or life-threatening (%)	361 (13.4)	47 (22.7)	19 (16.0)	295 (12.4)	** < 0.001**	361 (13.4)	227 (12.4)	122 (15.4)	**0.044**
Within 24 h after TAVI (%)	193 (7.1)	19 (9.2)	7 (5.9)	167 (7.0)	0.44	185 (7.0)	115 (6.3)	70 (8.8)	**0.024**
After 24 h after TAVI (%)	168 (6.2)	28 (13.5)	12 (10.1)	128 (5.4)	** < 0.001**	164 (6.2)	112 (6.1)	52 (6.5)	0.73
Acute renal failure (AKIN ≥ 2) (%)	124 (4.6)	24 (11.6)	0	100 (4.2)	** < 0.001**	128 (4.7)	79 (4.3)	42 (5.3)	0.32
Minor access-site complication (%)	259 (9.6)	20 (9.6)	9 (7.6)	230 (9.7)	0.74	259 (9.6)	190 (10.4)	61 (7.7)	**0.040**
Major access-site complication (%)	163 (6.0)	19 (9.1)	8 (6.7)	136 (5.7)	0.13	163 (6.0)	94 (5.1)	64 (8.1)	**0.0047**
Ascending aorta						160 (6.0)	147 (5.9)	13 (8.1)	0.34
Descending aorta						160 (6.0)	117 (5.8)	43 (6.8)	0.39
Infrarenal aorta/iliac						158 (6.0)	30 (4.3)	128 (6.6)	**0.030**
Femoral arteries						158 (6.0)	70 (5.0)	88 (7.2)	**0.022**
Permanent pacemaker implantation	409 (15.2)	25 (12.1)	30 (25.2)	354 (14.9)	**0.004**	399 (15.2)	268 (14.6)	131 (16.5)	0.25
Due to 3rd degree AV-block (%)	284 (70.5)	18 (72)	21 (70)	245 (70.4)	0.98	277 (70.5)	190 (72.5)	87 (66.4)	0.26
PVL ≥ mild (%)	113 (4.5)	8 (4.5)	3 (2.8)	102 (4.6)	0.68	112 (4.6)	80 (4.6)	32 (4.5)	0.92
Device success (%)	2518 (93.1)	188 (90.4)	111 (94.1)	2219 (93.3)	0.26	2451 (93.1)	1713 (93.4)	738 (92.6)	0.54
30-day mortality (%)	165 (6.3)	24 (11.6)	12 (10.5)	129 (5.6)	** < 0.001**	154 (5.7)	83 (4.5)	67 (8.4)	** < 0.001**

Values are *n* (%). None/mild calcification = VCSS ≤ 6, moderate/severe calcification = VCSS ≥ 7

*AKIN* acute kidney injury network, *AV* atrioventricular, *CKD* chronic kidney disease, *CTRL* control, *ESRD* end-stage renal disease, *LBBB* left bundle branch block, *PVL* paravalvular leak, *TAVI* transcatheter aortic valve implantation, *TIA* transient ischemic attack

Bold values indicate statistical significance as defined by *p* < 0.05

Moderate/severe vascular calcification was associated with a higher incidence of major access-site complications (8.0% vs. 5.1%, *p* = 0.0047) and major or life-threatening bleeding (15.4% vs. 12.4%, *p* = 0.044) compared to none/mild vascular calcification (Table 2). Major access complication rates were increased in patients with femoral artery calcification (none/mild vs. moderate/severe: 5.0 vs. 7.2%, *p* = 0.022) and infrarenal aortic/iliac calcification (4.3 vs. 6.6%, *p* = 0.030). While patients with moderate/severe vascular calcification showed similar event rates > 24 h after TAVI, an increased frequency of major/life-threatening bleeding was observed in the early post-procedural phase (< 24 h after TAVI: 6.3% vs. 8.8%, *p* = 0.024) compared to patients with none/mild vascular calcification. Acute renal failure, major stroke and myocardial infarction occurred at similar rates. A sub-analysis of patients with trans-femoral TAVI demonstrated that moderate/severe compared to none/mild vascular calcification was associated with higher rates of major access-site complications (8.0% vs. 5.0%, *p* = 0.023), 30-day mortality (6.7% vs. 4.2%, *p* = 0.046), and a trend for major or life-threatening bleeding (15.0% vs. 11.8%, *p* = 0.090, Online Resource 4).

### Survival

**Fig. 4:**
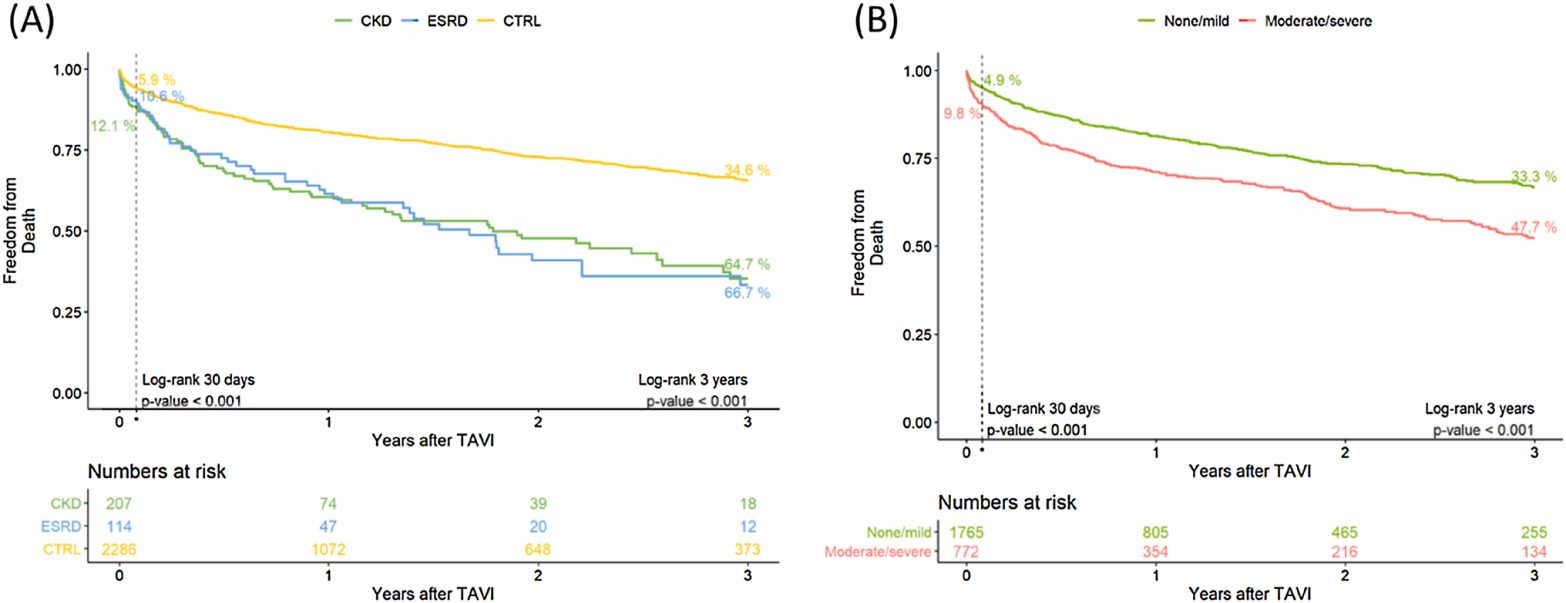
Kaplan–Meier survival up to 3 years after TAVI. **A** All-cause mortality according to renal function. **B** All-cause mortality according vascular calcification severity. *CKD* chronic kidney disease, *CTRL* control, *ESRD* end-stage renal disease, *TAVI* transcatheter aortic valve implantation. None/mild calcification = VCSS ≤ 6. Moderate/severe calcification = VCSS ≥ 7

Survival was impaired in patients with ESRD and CKD compared to CTRL at 30 days (89.4% and 87.9% vs. 94.1%), 1 year (61.4% and 60.5% vs. 80.5%), and 3 years (33.3% and 35.3% vs. 65.4%, all *p* < 0.001) after TAVI (Fig. 4A). Moderate/severe vascular calcification was associated with lower survival compared to none/mild calcification at 30 days (90.2% vs. 95.1%), at 1 year (71.1% vs. 81.3%), and 3 years (52.3% vs. 66.7%, all *p* < 0.001) after TAVI (Fig. 4B).

Multivariable analysis linked severely impaired LVEF, atrial fibrillation, NYHA class IV, anemia, severe pulmonary hypertension, moderate/severe vascular calcification, and non-trans-femoral access to a higher 30-day mortality (Table 3). At 3 years, ESRD, CKD, and diabetes were additionally associated with an increased risk for death.

**Table 3:** Multivariable analysis for mortality at 30 days and 3 years after TAVI

	30-day mortality after TAVI				3-year mortality after TAVI			
	Univariable HR (95% CI)	*p* value	Multivariable HR (95% CI)	*p* value	Univariable HR (95% CI)	*p* value	Multivariable HR (95% CI)	*p* value
Age	1.01 (0.99, 1.04)	0.28			1.00 (0.99, 1.01)	0.67		
Sex (male)	1.07 (0.77, 1.49)	0.70			1.28 (1.09, 1.52)	**0.0031**	1.17 (0.98, 1.39)	0.079
BMI	0.97 (0.94, 1.01)	0.13			0.99 (0.98, 1.01)	0.33		
Prior myocardial infarction	0.97 (0.60, 1.56)	0.90			1.14 (0.92, 1.42)	0.24		
Prior stroke	0.90 (0.56, 1.44)	0.66			1.25 (1.01, 1.54)	**0.043**	1.19 (0.96, 1.47)	0.12
LVEF < 30%	1.80 (1.17, 2.75)	**0.0071**	1.65 (1.07, 2.55)	**0.024**	1.87 (1.52, 2.31)	** < 0.001**	1.75 (1.41, 2.17)	** < 0.001**
Pulmonary hypertension (sPAP > 55 mmHg)	2.05 (1.38, 3.04)	** < 0.001**	1.75 (1.17, 2.60)	**0.0061**	1.50 (1.21, 1.87)	** < 0.001**	1.25 (1.01, 1.56)	**0.045**
CAD	1.13 (0.79, 1.61)	0.51			1.18 (0.99, 1.41)	0.067		
Diabetes	1.00 (0.70, 1.45)	0.98			1.38 (1.16, 1.64)	** < 0.001**	1.23 (1.03, 1.46)	**0.021**
Atrial fibrillation	2.07 (1.48, 2.88)	** < 0.001**	1.77 (1.26, 2.49)	** < 0.001**	2.06 (1.74, 2.43)	** < 0.001**	1.84 (1.56, 2.18)	** < 0.001**
Access: TF	0.48 (0.34, 0.67)	** < 0.001**	0.56 (0.38, 0.81)	**0.0023**	0.69 (0.58, 0.82)	** < 0.001**	0.74 (0.61, 0.90)	**0.0025**
NYHA IV	2.03 (1.37, 3.00)	** < 0.001**	1.57 (1.05, 2.36)	**0.029**	1.65 (1.34, 2.03)	** < 0.001**	1.26 (1.02, 1.56)	**0.033**
COPD	1.57 (1.07, 2.29)	**0.020**	1.33 (0.90, 1.96)	0.15	1.50 (1.24, 1.82)	** < 0.001**	1.41 (1.16, 1.71)	** < 0.001**
Anemia (Hb < 11)	1.86 (1.33, 2.60)	** < 0.001**	1.65 (1.17, 2.34)	**0.0048**	1.90 (1.60, 2.25)	** < 0.001**	1.65 (1.38, 1.98)	** < 0.001**
Prior CABG	1.12 (0.71, 1.76)	0.64			1.10 (0.88, 1.37)	0.40		
Moderate/severe calcification	2.10 (1.51, 2.94)	** < 0.001**	1.51 (1.04, 2.18)	**0.028**	1.64 (1.39, 1.94)	** < 0.001**	1.29 (1.07, 1.55)	**0.0081**
Renal function		0.18				** < 0.001**		
CKD	1.40 (0.81, 2.45)	0.23	1.18 (0.67, 2.08)	0.57	2.04 (1.57, 2.65)	** < 0.001**	1.79 (1.37, 2.34)	** < 0.001**
ESRD	1.71 (0.90, 3.27)	0.10	1.10 (0.57, 2.13)	0.78	2.39 (1.77, 3.22)	** < 0.001**	1.60 (1.18, 2.19)	**0.0028**

None/mild calcification = VCSS ≤ 6, moderate/severe calcification = VCSS ≥ 7

*BMI* body mass index, *CABG* coronary artery bypass grafting, *CAD* coronary artery disease, *CKD* chronic kidney disease, *COPD* chronic obstructive pulmonary disease, *ESRD* end-stage renal disease, *TAVI* transcatheter aortic valve implantation, *TF* transfemoral, *HR* hazard ratio, *LVEF* left-ventricular ejection fraction, *NYHA* New York Heart Association, *sPAP* systolic pulmonary artery pressure

Bold values indicate statistical significance as defined by *p* < 0.05

## Discussion

This retrospective analysis evaluated characteristics and outcomes of patients with CKD and ESRD undergoing TAVI. The main findings were: (i) Patients with ESRD on chronic hemodialysis or CKD represent a relevant group of patients referred for TAVI and constitute a particularly vulnerable patient cohort. (ii) Calcification of the aortic valve and device landing zone were similar but extensive vascular calcifications were observed more often in patients with CKD and particularly ESRD. (iii) Immediate procedural results were largely unaffected by severe renal impairment. (iv) Impaired renal function (CKD and ESRD) and extensive vascular calcifications were — among other comorbidities — independent predictors of impaired mid-term survival after TAVI.

The prevalence of ESRD and CKD was 4.4% and 7.7%, respectively, which was in line with previous reports [5–9, 11]. Furthermore, our results confirm that patients with impaired kidney function have a specific risk profile with many — particularly cardiovascular — comorbidities, known to play a substantial role in the progression of CKD, such as hypertension and diabetes associated with glomerulosclerosis [12]. Despite this increased risk profile, patients on ESRD were younger, reflecting the treatment allocation performed by the heart team and potentially a faster progression of AS severity in this group.

Impaired renal function is associated with severe calcifications of the coronary and peripheral arteries [15, 20]. Peripheral vascular calcifications constitute a potential risk for access-site complications and valvular calcification may impact procedural results [19, 21, 22]. Hence, these aspects are of particular importance in patients undergoing TAVI. To our knowledge this analysis represents the first systematic assessment of a combination of valvular and vascular calcification patterns in patients with AS and impaired kidney function. In line with previous findings, MDCT demonstrated similar valvular calcification patterns and severity for the AVC and LVOT in patients with and without impaired renal function that translated into similar prosthetic valve function [23]. In contrast, vascular calcium burden was significantly higher in patients with ESRD and — to a milder degree — CKD if compared to the control group. This was likely due to a dysregulation of the calcium, phosphate, and vitamin D metabolisms and secondary hyperparathyroidism [12–15]. Calcifications were located particularly in the infrarenal aorta/iliac and femoral arteries and were more pronounced in all segments except for the ascending aorta in patients with altered renal function. Interestingly, this did not translate into a higher rate of non-trans-femoral access for TAVI and our results suggest that severity but not distribution of vascular calcium is associated with renal impairment.

Procedural results and immediate clinical outcomes were similar in patients with and without severe renal impairment, even if on chronic hemodialysis. This included cerebrovascular events, myocardial infarctions, access-site complications and device success. Our results demonstrate that TAVI is a safe and effective option in patients with severe AS, irrespective of kidney function, and may define a particular role for TAVI in these patients.

While early procedure-related bleedings were similar, bleeding unrelated to TAVI was more frequent in patients with CKD and ESRD. Impaired kidney function was linked to increased bleeding risks and blood transfusions before, likely due to a higher prevalence of anemia and altered calcium metabolism. Platelet dysfunction and hypercoagulability may also contribute [8, 10, 24, 25]. Baseline anemia was identified as an independent predictor of 30-day and 3-year mortality providing further evidence as an important risk factor in patients with renal impairment. Unsurprisingly, patients with CKD had the highest incidence of post-procedural AKI with an elevated risk for a faster progression of their underlying disease potentially leading to ESRD [26].

Moreover, we observed a higher rate of conduction disturbances yielding pacemaker implantation in patients with ESRD. This was in accordance with previous studies. Vascular calcifications, prior MI and impaired left ventricular function were reported as risk factors, all of which were more prevalent in our ESRD patients. Myocardial fibrosis and hemodialysis, that is associated with volume shifts and electrolyte changes, may be additional drivers. Albeit similar in this analysis, calcification patterns of the AVC and LVOT and implanted THV types are well-known risk factors for conduction disturbances after TAVI [27]. These aspects should be considered when planning TAVI in patients with kidney disease deemed at increased risk for the development of conduction disturbances [10, 12, 24, 28].

Despite low early complication rates, short-term mortality was significantly higher in patients with ESRD and CKD, largely driven by cardiovascular comorbidities and impaired renal function. Elevated short-term mortality in these patients has been reported before, ranging from 6 to 24% for ESRD and 6 to 15% for CKD at 30 days and with increased operative mortality in case of SAVR [5, 9–11, 16, 29, 30].

For mid-term outcomes, multivariable analysis identified impaired renal function (ESRD and CKD), moderate/severe vascular calcifications and cardiovascular comorbidities as independent predictors of 3-year-mortality. Although the majority of ESRD patients were male, we did not find an independent association of gender and outcome in our analysis. Others have investigated the effect of impaired renal function on mid-term mortality before but the impact of vascular calcification in this patient population is novel and warrants further investigation [5, 9, 11, 16, 29, 31]. Congruent with early studies on vascular calcifications and access-site complications, we found patients with extensive vascular calcifications at higher risk for procedure-associated bleeding, major access complications, and 30-day mortality [21, 22]. Correspondingly, access complications were mainly driven by calcification of the infrarenal aorta/iliac and femoral arteries. Beyond that, it remains to be determined whether overall calcium burden serves as surrogate parameter of underlying disease, or a unique risk factor itself. In any case, our results highlight the importance of assessing the vascular calcium burden during pre-TAVI workup.

Early structural valve deterioration (SVD) in patients with ESRD remains a concern after SAVR and a recent meta-analysis even favored mechanical over biological prostheses in these patients [32, 33]. Corresponding durability data for THV are unavailable at current and are urgently needed to define the role of TAVI in younger patients with impaired renal function or hemodialysis. Unfortunately, limited follow-up did not provide sufficient information on this aspect in our analysis. Further limitations relate to the retrospective single-center design. Results are only hypothesis-generating. The analysis covered a time frame of 8 years which may explain an overall increased operative risk and complication rate. Differences in group sizes, determined by the prevalence of CKD and ESRD, complicate the interpretation of results. Furthermore, stratification according to renal function was performed at baseline only and the duration of chronic hemodialysis was unavailable although of potential importance. In the absence of standardized methods to evaluate vascular calcification patterns from contrast-enhanced MDCT, we defined a pragmatic approach to assess vascular calcification burden in a semi-quantitative fashion and according to location. As valvular calcification was assessed in contrast-enhanced MDCT images, the Agatson-Score was not applicable in our analysis.

## Conclusion

Patients with ESRD on chronic hemodialysis and CKD constitute a particularly vulnerable patient group with extensive vascular calcifications. Procedural results and early clinical outcomes were, except for elevated rates of late bleeding and conduction disturbances yielding pacemaker implantation, similar to patients with preserved renal function, yielding TAVI a particularly valuable treatment option in these high-risk operative patients. Impaired mid-term survival likely reflects the underlying renal disease in conjunction with other cardiovascular comorbidities. Vascular calcification burden was identified as a novel risk marker for impaired survival and procedural complications, warranting further investigation and meticulous planning of TAVI procedures. Longer follow-up after TAVI is now required to evaluate the durability and rule out early prosthetic degeneration in younger patients with ESRD and CKD.

## Supplementary Information

Below is the link to the electronic supplementary material.

Supplementary file1 (DOCX 72 KB)

## Abbreviations

AS: Aortic stenosis
AVC: Aortic valve calcification
CKD: Chronic kidney disease
CTRL: Control group
ESRD: End-stage renal disease
GFR: Glomerular filtration rate
HR: Hazard ratio
LVEF: Left-ventricular ejection fraction
LVOT: Left-ventricular outflow tract
MDCT: Multi-detector computed tomography
PM: Pacemaker
TAVI: Transcatheter aortic valve implantation
VCSS: Vascular calcification severity score
