# Supplementary material for: End-stage renal disease, calcification patterns and clinical outcomes after TAVI
Source: Clin Res Cardiol. 2021 Nov 13;111(12):1313–24. doi: 10.1007/s00392-021-01968-y (PMC9681684; doi:10.1007/s00392-021-01968-y)
Supplement: Supplementary file 1 — Supplementary file1 (DOCX 72 KB) [file 392_2021_1968_MOESM1_ESM.docx]

**Supplementary information**

**Online Resource 1:** **Baseline characteristics according to vascular calcification severity**

|  | All (N=2639) | None/mild (N=1842) | Moderate/severe (N=797) | p-value |
| --- | --- | --- | --- | --- |
| Sex (male) (%) | 1250 (47.4) | 798 (43.3) | 452 (56.7) | **<0.001** |
| Age (years) | 81.0 (76.6, 84.8) | 81.1 (76.5, 84.7) | 80.8 (76.7, 85.2) | 0.48 |
| BMI (kg/m²) | 26.2 (23.4, 29.7) | 26.4 (23.5, 30.1) | 25.7 (23.3, 28.7) | **<0.001** |
| NYHA class IV (%) | 337 (13.0) | 223 (12.3) | 114 (14.6) | 0.12 |
| LVEF <30% (%) | 283 (11.0) | 190 (10.5) | 93 (12.0) | 0.31 |
| Mean transvalvular gradient (mmHg) | 33.0 (23.0, 44.0) | 34.0 (24.0, 46.0) | 30.0 (21.0, 41.0) | **<0.001** |
| Effective orifice area (cm2) | 0.8 (0.6, 0.9) | 0.8 (0.6, 0.9) | 0.8 (0.6, 0.9) | 0.42 |
| Mitral regurgitation ≥moderate (%) | 852 (33.2) | 571 (31.8) | 281 (36.4) | **0.029** |
| Tricuspid regurgitation ≥moderate (%) | 596 (24.0) | 413 (23.6) | 183 (24.9) | 0.53 |
| Pulmonary hypertension  (PAP syst >55 mmHg (%)) | 339 (12.8) | 231 (12.5) | 108 (13.6) | 0.52 |
| Diabetes mellitus (%) | 761 (28.9) | 515 (28.0) | 246 (30.9) | 0.13 |
| Atrial fibrillation (%) | 769 (30.1) | 518 (29.1) | 251 (32.3) | 0.12 |
| PAD (%) | 765 (29.0) | 385 (20.9) | 380 (47.7) | **<0.001** |
| Dialysis prior to TAVI (%) | 116 (4.4) | 59 (3.2) | 57 (7.2) | **<0.001** |
| Prior PM / ICD (%) | 281 (10.7) | 188 (10.2) | 93 (11.7) | 0.29 |
| Coronary artery disease (%) | 1695 (64.6) | 1064 (58.2) | 631 (79.4) | **<0.001** |
| Prior myocardial infarction (%) | 373 (14.1) | 218 (11.8) | 155 (19.4) | **<0.001** |
| Prior PCI (%) | 968 (36.8) | 592 (32.2) | 376 (47.5) | **<0.001** |
| Prior CABG (%) | 372 (14.1) | 184 (10.0) | 188 (23.7) | **<0.001** |
| Prior stroke (%) | 396 (15.0) | 259 (14.1) | 137 (17.2) | **0.045** |
| COPD (%) | 494 (18.7) | 312 (16.9) | 182 (22.9) | **<0.001** |
| Anemia (hemoglobin <11 g/dl) (%) | 766 (29.1) | 492 (26.8) | 274 (34.5) | **<0.001** |
| GFR (CKD-EPI) (mL/min/1.73m²) | 57.1 (41.1, 74.2) | 58.4 (43.2, 75.3) | 54.4 (37.1, 71.1) | **<0.001** |
| STS PROM (%) | 4.2 (2.7, 6.4) | 3.9 (2.6, 5.8) | 5.1 (3.1, 8.2) | **<0.001** |
| Logistic Euro-Score II (%) | 4.4 (2.5, 7.6) | 3.9 (2.4, 6.5) | 5.8 (3.4, 10.1) | **<0.001** |

Values are n (%) or median (interquartile range).

BMI: body mass index, CABG: coronary artery bypass grafting, CAD: coronary artery disease, COPD: chronic obstructive pulmonary disease, GFR: glomerular filtration rate, ICD: implantable cardioverter defibrillator, LVEF: left-ventricular ejection fraction, NYHA: New York Heart Association, PAD: peripheral artery disease, PAP: pulmonary artery pressure, PCI: percutaneous coronary interventions, PM: pacemaker, STS-PROM: Society of Thoracic Surgeons Predicted Risk of Mortality

**Online Resource 2: Baseline MDCT-based measurements**

|  | All  (N=2712) | CKD  (N=210) | ESRD  (N=119) | CTRL  (N=2383) | p-value |
| --- | --- | --- | --- | --- | --- |
| Aortic valve and root calcification and geometry | | | | | |
| AVC calcium (mm^3^) | 474.1  (255.3, 815.8) | 440.5  (211.0, 801.3) | 395.8  (248.0, 679.3) | 480.9  (256.2, 827.3) | 0.17 |
| AVC LC (mm^3^) | 114.2  (49.5, 239.3) | 106.4  (40.6, 232.1) | 104.6  (47.5, 189.5) | 115.6  (51.3, 246.9) | 0.13 |
| AVC NC (mm^3^) | 200.0  (96.3, 354.9) | 176.4  (56.7, 320.9) | 171.9  (62.6, 340.1) | 202.2  (99.9, 359.1) | **0.044** |
| AVC RC (mm^3^) | 119.0  (52.6, 229.0) | 118.3  (51.0, 227.2) | 126.6  (57.9, 191.3) | 119.0  (52.3, 231.2) | 0.87 |
| LVOT calcium (mm^3^) | 1.9 (0, 33.1) | 0.9 (0, 39.5) | 1.6 (0, 14.2) | 2.0 (0, 33.8) | 0.50 |
| Annulus perimeter (mm) | 77.5  (72.2, 83.0) | 76.5  (72.9, 81.0) | 81.3  (75.7, 84.5) | 77.5  (72.1, 82.8) | **0.001** |
| LVOT perimeter (mm) | 79.1  (73.2, 85.4) | 76.8  (71.9, 84.5) | 82.5  (76.4, 87.6) | 79.1  (73.0, 85.2) | **0.006** |
| STJ perimeter (mm) | 90.5  (83.9, 98.0) | 89.5  (83.7, 96.8) | 93.1  (87.6, 99.3) | 90.4  (83.8, 98.0) | 0.16 |
| LCA height (mm) | 14.3  (12.2, 16.5) | 14.6  (12.1, 16.3) | 15.8  (13.0, 17.8) | 14.2  (12.1, 16.4) | **0.010** |
| RCA height (mm) | 16.9  (14.7, 19.2) | 16.7  (14.5, 19.4) | 17.6  (15.8, 20.3) | 16.9  (14.7, 19.1) | 0.20 |
| Vascular calcification and distribution | | | | | |
| CFA diameter (mm) | 7.0 (6.0, 8.0) | 7.0 (6.0, 8.0) | 7.0 (6.0, 8.0) | 7.0 (6.0, 8.0) | 0.21 |
| Overall: |  |  |  |  | **<0.01** |
| None/mild (%) | 1842 (69.8) | 129 (66.2) | 59 (50.9) | 1654 (71.0) |  |
| Moderate/severe (%) | 797 (30.2) | 66 (33.9) | 57 (49.1) | 674 (28.95) |  |
| Ascending Aorta: |  |  |  |  | 0.23 |
| None/mild (%) | 2497 (93.9) | 181 (91.4) | 109 (92.4) | 2207 (94.2) |  |
| Moderate/severe (%) | 162 (6.1) | 17 (8.6) | 9 (7.6) | 136 (5.8) |  |
| Descending Aorta: |  |  |  |  | **<0.01** |
| None/mild (%) | 2028 (76.2) | 148 (74.8) | 72 (61.0) | 1808 (77.1) |  |
| Moderate/severe (%) | 632 (23.8) | 50 (25.3) | 46 (38.98) | 536 (22.9) |  |
| Infrarenal Aorta/Iliac: |  |  |  |  | **0.024** |
| None/mild (%) | 709 (26.8) | 44 (22.1) | 21 (18.1) | 644 (27.62 |  |
| Moderate/severe (%) | 1938 (73.2) | 155 (77.9) | 95 (81.9) | 1688 (72.4) |  |
| Femoral arteries: |  |  |  |  | **<0.01** |
| None/mild (%) | 1415 (53.5) | 101 (50.8) | 37 (31.9) | 1277 (54.8) |  |
| Moderate/severe (%) | 1230 (46.5) | 98 (49.3) | 79 (68.1) | 1053 (45.2) |  |

Values are n (%) or median (interquartile range).

AVC: aortic valve calcification, CFA: common femoral artery, CKD: chronic kidney disease, CTRL: Control, ESRD: end-stage renal disease, LC: left coronary, LCA: left coronary artery, LVOT: left ventricular outflow tract, MDCT: multi-detector computed tomography, NC: noncoronary, RC: right coronary, RCA: right coronary artery, STJ. sinotubular junction, VCSS: vascular calcification severity score

None/mild calcification = VCSS ≤6,

Moderate/severe calcification = VCSS ≥7

**Online Resource 3: Baseline THV types**

|  | All (N=2,712) | CKD (N=210) | ESRD (N=119) | CTRL (N=2,383) | p-value |
| --- | --- | --- | --- | --- | --- |
| Balloon-expandable (%) | 1093 (40.3) | 88 (41.9) | 42 (35.3) | 963 (40.4) | 0.48 |
| Sapien XT (%) | 250 (9.0) | 19 (9.0) | 6 (5.0) | 225 (9.2) |  |
| Sapien 3 (%) | 843 (31.1) | 69 (32.9) | 36 (30.3) | 738 (31.0) |  |
| Mechanically- and self-expanding (%) | 1619 (59.7) | 122 (58.1) | 77 (64.7) | 1420 (59.6) | 0.48 |
| CoreValve Evolut/Evolut R (%) | 337 (12.4) | 27 (12.9) | 16 (13.5) | 294 (12.4) |  |
| JenaValve (%) | 153 (5.6) | 11 (5.2) | 7 (5.9) | 135 (5.7) |  |
| Acurate/Acurate Neo (%) | 627 (23.1) | 55 (26.2) | 32 (26.9) | 540 (22.7) |  |
| Lotus (%) | 117 (4.3) | 3 (1.4) | 8 (6.7) | 106 (4.4) |  |
| Portico (%) | 156 (5.8) | 10 (4.8) | 4 (3.4) | 142 (6.0) |  |
| Others (%) | 229 (8.4) | 16 (7.8) | 10 (8.4) | 203 (8.4) |  |

Values are n (%).

CKD: chronic kidney disease, CTRL: Control, ESRD: end-stage renal disease, THV: transcatheter heart valve

**Online Resource 4:** **Procedural complications and 30-day outcomes after TAVI through transfemoral access according to vascular calcification severity**

|  | All (N=2037) | None/mild (N=1609) | Moderate/severe (N=428) | p-value |
| --- | --- | --- | --- | --- |
| Myocardial infarction (%) | 23 (1.1) | 19 (1.2) | 4 (0.9) | 0.86 |
| Any stroke/TIA (%) | 69 (3.4) | 59 (3.7) | 10 (2.3) | 0.23 |
| Disabling Stroke | 32 (1.6) | 29 (1.8) | 3 (0.7) | 0.16 |
| Major or life-threatening bleeding (%) | 253 (12.5) | 189 (11.8) | 64 (15.0) | 0.090 |
| within 24h after TAVI (%) | 131 (6.5) | 95 (5.9) | 36 (8.4) | 0.078 |
| after 24h after TAVI (%) | 122 (6.0) | 94 (5.9) | 28 (6.6) | 0.67 |
| Acute renal failure (AKIN ≥ 2) (%) | 69 (3.4) | 56 (3.5) | 13 (3.0) | 0.76 |
| Access site complications: |  |  |  |  |
| Minor access site complication (%) | 225 (11.1) | 179 (11.2) | 46 (10.8) | 0.91 |
| Major access site complication (%) | 114 (5.6) | 80 (5.0) | 34 (8.0) | **0.023** |
| Permanent pacemaker implantation | 300 (14.8) | 233 (14.6) | 67 (15.7) | 0.73 |
| PVL ≥mild (%) | 97 (5.1) | 73 (4.8) | 24 (6.0) | 0.39 |
| Device success (%) | 1906 (93.8) | 1508 (94.1) | 398 (93.0) | 0.47 |
| 30-day mortality (%) | 91 (4.7) | 64 (4.2) | 27 (6.7) | **0.046** |

Values are n (%) or median (interquartile range).

AKIN: Acute Kidney Injury Network, PVL: paravalvular leak, TAVI: transcatheter aortic valve implantation, TIA: transient ischemic attack

None/mild calcification = VCSS ≤6,

Moderate/severe calcification = VCSS ≥7
